# Laboratory Dont’s

**Published:** 1914-04

**Authors:** 


### ABSTRACTS

LABORATORY DONT'S, *The Laboratory News*, January, 1914. Don't expect the Laboratory *alone* to make the diagnosis, prognosis and suggest all the treatment of a case. The laboratory does not pretend to push aside clinical symptomatology.

Don't abandon a diagnosis of pulmonary tuberculosis because you fail to find tubercle bacilli in the sputum. Remember that a positive finding of tubercle bacilli is a late diagnosis, not an early one, of pulmonary tuberculosis.

Don't forget that hoarseness of the voice, without any other good reason is suggestive of laryngeal tuberculosis. In such cases you ought to find tubercle bacilli early in the process.

Don't rely on the microscopical evidence of renal tuberculosis. Remember that in renal tuberculosis the positive diagnosis can best be made by inoculating a few guinea pigs with urinary sediment and watching them develop tuberculosis.

Don't rely on the Von Pirquet skin test for tuberculosis except in children. Remember that many of us have passed through and recovered from incipient tuberculosis without ever having known it. Such cases later on in life if tested will give a positive Von Pirquet Test. A negative Von Pirquet in an adult is usually very significant in that the individual never has had tuberculosis and does not have it now. A positive Von Pirquet may mean that he has had tuberculosis or that he has it now. It does not differentiate between a past and a present tubercular infection.

Don't use bacterial vaccines unless you know the nature of the offending microbe.

Don't use biological products unless you are acquainted with the rationale of their preparation and action.

Don't expect to cure rabies once the symptoms have developed. Remember that the time to administer preventive treatment is any time within ten days after the infliction of the dog-bite.

Don't kill the dog when he bites a patient. Keep him under observation until he develops rabies. There will still be ample time to administer the Pasteur treatment. You may find the Negri bodies in the brain of the dog if he is killed right away, but the chances are, you will not. The incubative period in man is seldom under three weeks.

Don't make a positive diagnosis of gonorrhea in the female unless you use the blood complement fixation test. There are many cases of uterine discharge which are not gonorrheal. There is no absolute method of identification of the gonococcus even with the Gram stain, unless you can isolate it in pure culture. This is difficult to carry out in uterine or vaginal discharges because of the multitude of other bacteria present which may prevent you from obtaining a pure culture.

Don't swear a man's liberty away in a case of suspected rape from the mere presence of a discharge in the alleged victim. Be sure it is gonorrheal before you say so. The microscope alone in such a case is insufficient evidence.

